# Quantification of myocardial scar assessed by late gadolinium enhancement CMR in the multi-ethnics study of atherosclerosis: comparisons of 7 different methods

**DOI:** 10.1186/1532-429X-15-S1-O49

**Published:** 2013-01-30

**Authors:** Patricia B Rizzi, Marcelo Nacif, Gustavo J Volpe, Evrim B Turkbey, Bharath Ambale Venkatesh, Rob J van der Geest, David A Bluemke, Joao A Lima

**Affiliations:** 1 Cardiology, Jonhs Hopkins University School of Medicine, Baltimore, MD, USA; 2 Radiology and Imaging Sciences, National Institutes of Health Clinical Center, Bethesda, MD, USA; 3 Department of cardiac Imaging, Clinica de Diagnóstico por Imagem (CDPI), Rio de Janeiro, Brazil; 4 Department of Radiology, Division of Image Processing, Leiden University Medical Center, Leiden, the Netherlands

## Background

Late gadolinium enhancement cardiac magnetic resonance (LGE-CMR) is the non-invasive reference standard for myocardial scar assessment and has prognostic value for ischemic and non-ischemic cardiomyopathies. However, actual standard methods for determining scar quantification compromise its reproducibility. This study evaluates reliability of seven techniques on scar quantification in a large multi-center study.

## Methods

1666 participants of the Multi-Ethnic Study of Atherosclerosis, age range 55-94 yrs, underwent a LGE-CMR study using 1.5T Tesla Siemens or GE scanners at six centers. Myocardial scar was visually detected in 137 studies (48% ischemic scar). The reference standard for quantitative analysis was semi-automated, based on choosing by visual inspection the best computed assisted planimetry, delineated using different automatic thresholds, with subsequent manual correction of partial volume and artifacts. This was compared to 6 different automatic methods including thresholding by 2, 4, 6 or 8 standard deviations (SD) above mean remote myocardial signal intensity (SI), full with half maximum (FWHM) and background correction (BCT) techniques. The BCT computes the threshold for each slice individually based on the sum of the mean SI of the entire myocardium, 2SD of the remote myocardium and 2SD of a ROI placed in the air. Inter/intraobserver agreement and reproducibility in studies of different scanners were assessed by paired t-test, concordance correlation coefficient (CCC) and Bland-Altman analysis.

## Results

**Figure 1:**
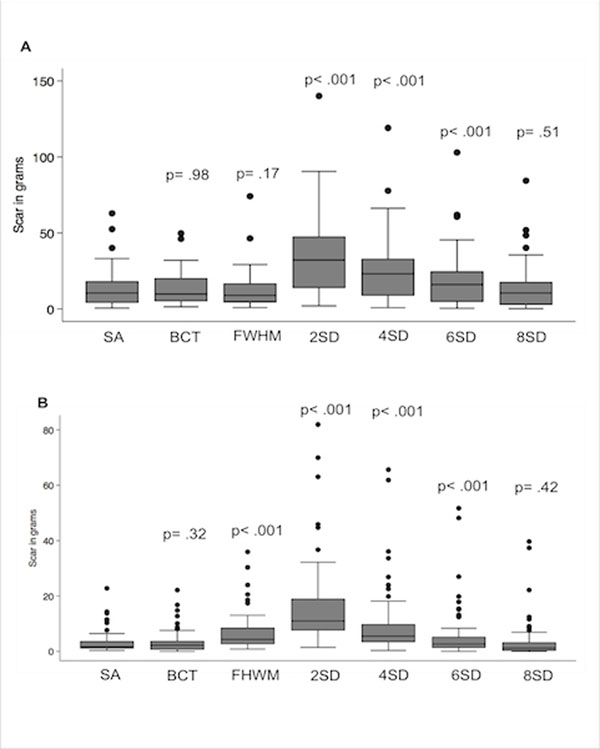
Box plots showing the variability in the mean amount of scar in grams depending on the quantitative method used according to the scar pattern: ischemic (A) and non-ischemic (B). The p-values were derived from the t-test for differences in mean amount of scar between each automatic method and the semi-automated technique. In the ischemic group (A) BCT, FWHM and 8SD were not significantly different from the SA, while in the non-ischemic group (B), only BCT and 8SD did not differ statistically from the SA. SA = semi-automated; BCT = background correction technique; FWHM = full width at half maximum; SD = standard deviation

The mean amount of scarred myocardium in grams varied substantially between methods (figure 1). There was no significant difference between the reference and 8-SD, FWHM or BCT techniques in the ischemic group (p = 0.98, 0.17 and 0.51; respectively); and between the semi-automated and 8-SD or BCT in non-ischemic cases (p = 0.32 and 0.42, respectively). These results were similar when comparing agreement to the semi-automated method in studies from different scanners, but less bias and narrower limits of agreement were observed for BCT and FWHM (table 1). The 2SD, 4SD and 6SD overestimated the amount of scar by factors of 2.6, 1.8 and 1.3 in ischemic and in 5.2, 3.1 and 1.9 in non-ischemic cases. Also, the FWHM overestimated scar by 2.2 times (p< 0.001) in this last group. All methods had excellent reproducibility, the CCC for intra and inter-reader reproducibility varied from 0.97 (95%CI=0.95, 1.0) to 0.99 (95%CI= 0.97, 1.0).

**Table 1:** Reproducibility according to type of MRI scanner

			Scan Type			
	
			Siemens (n = 109)		GE (n = 23)	
	
			Bias	95% loa	Bias	95% loa
Method of Quantification by Scar Pattern	Ischemic	BCT	-0.27	-11.8, 11.2	0.96	-5.7, 7.6
	
		FWHM	0.98	-8.5, 10.4	0.31	-8.1, 8.8
	
		8SD	-0.09	-12.4, 12.2	2.8	-5.1, 10.7
	
	
	
	Non-Ischemic	BCT	-0.24	-3.2, 2.7	0.17	-2.5, 2.8
		
		8 SD	-0.67	-9.3, 8.3	1.1	-1.9, 4.1




Comparison of Bland Altman analysis for average difference (bias) and the limits of agreement between the semi-automated method and three automatic techniques according to the scanner type where they were performed. Only the three automatic techniques that did not show significant difference from the semi-automated in the overall analysis were included in this subanalysis. BCT= Background corrected technique, 8 SD= 8 standard deviations, FWHM= Full Width at Half Maximum

## Conclusions

In the setting of a multi-center trial, BCT and FHWM methods are the preferred methods for automated quantification for ischemic scar, while only BCT is recommended for non-ischemic scar.

## Funding

This study was supported by the National Heart, Lung, and Blood Institute grant (RO1-HL66075-01) and the MESA study contracts NO1-HC-9808, NO1-HC-95168, and NO1-HC-95169.

